# Closing the Exposure Dimension Gap in Chronic Disease Prevention: Evidence from Per- and Polyfluoroalkyl Substances and the Global Burden of Diabetic Kidney Disease

**DOI:** 10.34133/hds.0479

**Published:** 2026-08-19

**Authors:** Ya Gao, Changsheng Guo, Jian Xu, Fengchang Wu

**Affiliations:** ^1^ College of Water Sciences, Beijing Normal University, Beijing 100875, China.; ^2^ State Key Laboratory of Environmental Criteria and Risk Assessment, Chinese Research Academy of Environmental Sciences, Beijing 100012, China.

## Introduction

Chronic disease prevention has long been organized around biomedical and behavioral risks, including obesity, hypertension, dyslipidemia, glycemic control, diet, and physical activity [1,2]. These strategies remain central to diabetes care, yet the global burden of diabetes and its renal complications continues to rise [3,4]. This persistent burden suggests that current prevention frameworks may not fully capture upstream determinants of metabolic and kidney disease risk. Environmental exposure remains poorly integrated into this framework. Persistent chemicals may influence metabolic and renal risk through endocrine disruption, oxidative stress, altered lipid and glucose metabolism, and changes in renal transport and elimination [5–8]. We refer to the weak translation of exposure evidence into chronic disease surveillance, burden assessment, and prevention policy as the exposure dimension gap.

Global burden assessment frameworks, particularly the Global Burden of Disease (GBD) studies, have incorporated several established environmental risks, including air pollution, unsafe water and sanitation, lead exposure, and occupational hazards [9,10]. However, many emerging chemical exposures remain insufficiently characterized because of limited exposure data and incomplete integration into disease burden models. Per- and polyfluoroalkyl substances (PFASs) provide a useful case for examining this gap because they are persistent, widely detected in human populations [11] and increasingly relevant to discussions of metabolic dysfunction and kidney function changes [12,13]. Diabetic kidney disease (DKD) represents the intersection of long-term metabolic injury, progressive kidney dysfunction, disability, and premature mortality, providing an integrated outcome for evaluating how environmental exposures may contribute to chronic disease burden and prevention opportunities [14,15].

China offers a relevant case within this global problem: A large diabetes population, substantial DKD burden, and documented PFAS exposure coexist with growing demand for prevention-oriented environmental health policy. This perspective uses PFAS and DKD to examine how exposure evidence can be connected with chronic disease burden and policy action. The aim is not to treat PFAS as the sole driver of DKD but to show why exposure surveillance should become part of chronic disease prevention and how pollution control may generate measurable health benefits.

## PFAS Exposure as an Entry Point to Chronic Disease Prevention

PFAS exposure remains widespread despite restrictions on several legacy compounds. These chemicals are routinely detected in human serum, drinking water, and food chains and can persist in ecosystems and human populations long after release [16]. Replacement chemicals and precursor forms are still incompletely monitored, leaving important exposure profiles insufficiently characterized. Monitoring studies suggest that PFAS contamination is broader than current surveillance captures, with frequent exceedance of drinking water guidelines even in regions without obvious point sources [17]. In the United States, National Health and Nutrition Examination Survey data from 1999 to 2020 showed marked declines in serum perfluorooctane sulfonate (PFOS) and perfluorooctanoic acid after production phaseouts, yet more than 96% of individuals still had detectable concentrations [11]. In China, serum PFOS and perfluorooctanoic acid levels remain substantially higher in some regions [18], showing that PFAS exposure is both persistent and unevenly distributed.

Experimental studies have reported renal vascular congestion, interstitial oedema, altered endothelial permeability, impaired filtration, and early tubular damage after PFAS exposure [19]. These findings support biological links with metabolic disease and renal complications, including DKD. Population-based studies have linked serum PFAS concentrations with kidney function, but reported patterns vary, including inverse associations and nonlinear relationships with estimated glomerular filtration rate [20]. Causal interpretation is further complicated by PFAS toxicokinetics, since kidney function can influence PFAS elimination, tubular secretion, and reabsorption. The central challenge is not to attribute DKD burden to a single chemical pathway but to determine how persistent chemical exposures can be monitored, evaluated, and connected with burden assessment and policy action.

## Diabetes and DKD Burden as a Case for Exposure-Informed Prevention

GBD estimates describe the distribution and trends of diabetes and DKD burden.

Our previous study used serum PFOS concentrations from 9 Chinese provinces, together with the relationship between PFOS exposure and diabetes risk derived from a meta-analysis. We applied comparative risk assessment to quantify the diabetes burden attributable to PFOS exposure. The results showed that this burden reached an estimated 6.84 million cases in 2018, with substantial differences across provinces and demographic groups [21].

Globally, the age-standardized disability adjusted life year (DALY) rate for diabetes increased from 663.11 per 100,000 population in 1990 to 916.25 per 100,000 in 2021, with incidence continuing to rise worldwide and in China [4]. In China, DKD accounted for 71.9% of diabetes deaths, 29.2% of DALYs, and 9.0% of total diabetes cases in 2021. DKD burden showed geographical heterogeneity and socioeconomic gradients, with China carrying a higher burden than would be expected from development level alone.

**Fig. 1.**
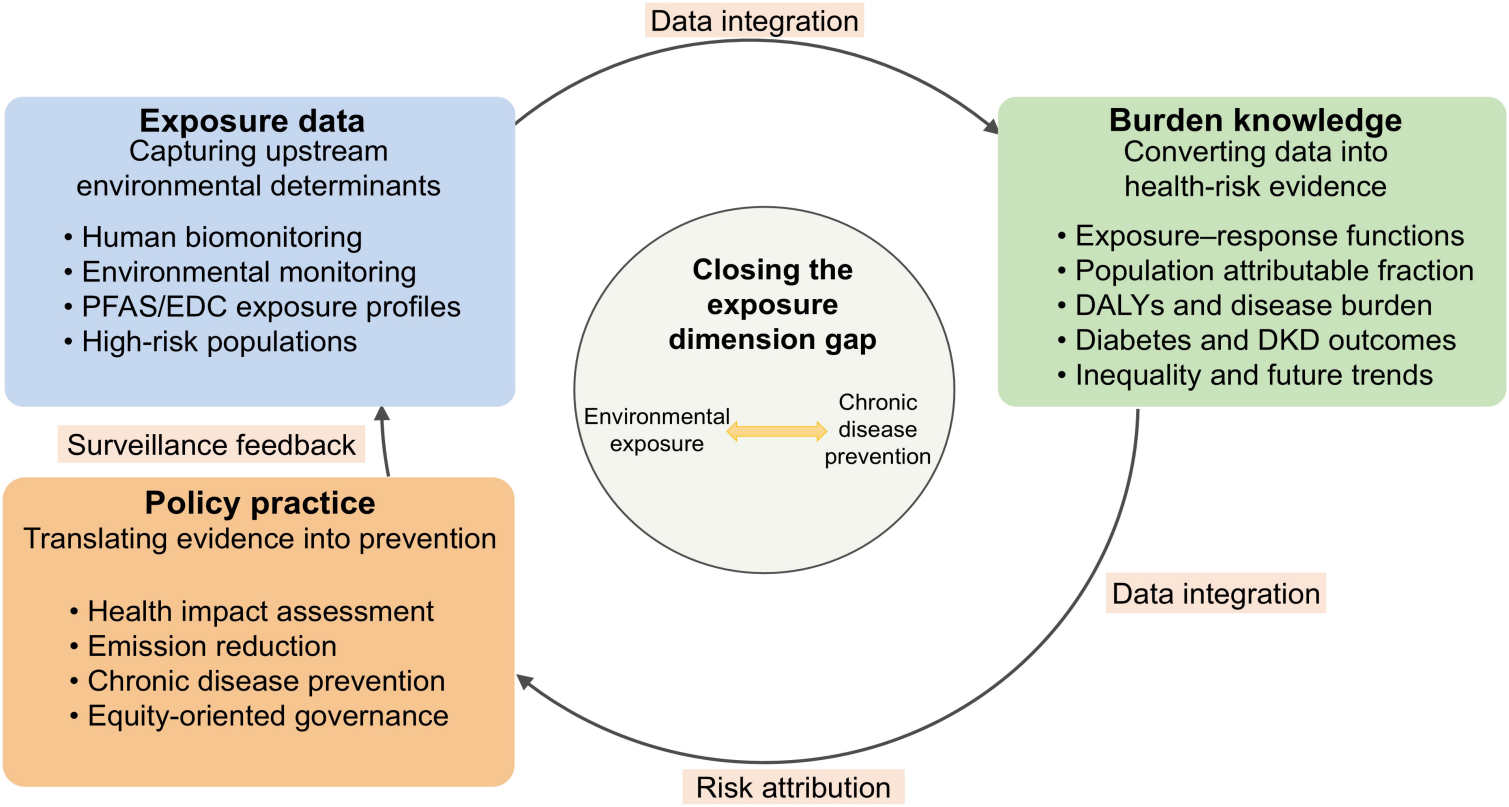
Exposure–burden–policy cycle for closing the exposure dimension gap in chronic disease prevention. PFAS, per- and polyfluoroalkyl substances; EDC, endocrine disrupting chemical; DALYs, disability adjusted life years; DKD, diabetic kidney disease.

Burden estimates identify where diabetes and DKD are rising but cannot indicate which upstream exposures may contribute to those patterns. Biomonitoring and environmental monitoring can define exposure patterns, disease registries can provide outcome information, and exposure–response evidence can support population attributable fraction and DALY estimation. Together with inequality metrics and forecasting models, these data can move PFAS evidence from scattered observations toward policy-relevant burden assessment. This linkage forms the basis of an exposure–burden–policy cycle, in which surveillance identifies upstream risks, burden assessment estimates their health relevance, and policy feedback evaluates whether exposure reduction produces measurable health gains. Figure 1 summarizes this cycle and shows how exposure surveillance, burden quantification, policy action, and evaluation can operate as a connected framework.

## From Burden Knowledge to Policy Practice

For chronic disease prevention, exposure evidence becomes useful only when it can guide surveillance, risk attribution, and policy evaluation. Diabetes and kidney disease are already priorities under the “Healthy China 2030” initiative, but environmental exposure surveillance and chemical risk assessment remain largely separate from chronic disease strategies. This separation limits the ability to identify modifiable upstream drivers, quantify health gains from pollution control, or target prevention in high-risk regions. Closing the exposure dimension gap requires exposure, disease burden, and prevention targets to be assessed within the same policy framework.

Biomonitoring, environmental monitoring, and health outcome data could be linked through interoperable databases [22]. Longitudinal health cohorts such as the China Health and Nutrition Survey, together with environmental monitoring networks and disease registries, could provide complementary data streams for exposure-informed prevention. China Health and Nutrition Survey captures changes in diet, socioeconomic conditions, and health status over time; environmental monitoring can identify PFAS and other endocrine-disrupting chemicals in relevant media; and disease registries can provide outcome information on diabetes, DKD, renal function, and mortality. Repeated biomonitoring would help address exposure timing, cumulative exposure, and disease latency, which are central to chronic disease interpretation. Because PFAS exposure rarely occurs in isolation, mixture assessment should also be incorporated into this system.

Evidence on exposure–response relationships, together with population attributable fractions and DALYs, can quantify the health gains from pollution control, particularly in provinces with high PFAS exposure or fluorochemical production. A joint roadmap developed by environmental, health, and industrial authorities could align data standards, assessment tools, and policy objectives. Equity indicators would help identify regions where high exposure and limited socioeconomic capacity overlap. International frameworks, such as the European Union’s Regulation on the Registration, Evaluation, Authorisation and Restriction of Chemicals regulation and the US Environmental Protection Agency’s PFAS Strategic Roadmap, offer useful references for monitoring, risk assessment, and phased chemical control.

## Conclusion

The rising burden of diabetes and DKD shows why chronic disease prevention needs to look beyond clinical management and individual behavior. PFAS exposure not only is an environmental contamination issue but also illustrates how persistent chemical exposures may be overlooked as upstream environmental determinants of chronic disease risk. Diabetes and DKD will be harder to control if exposure evidence remains outside routine surveillance, risk assessment, and burden evaluation. A more connected evidence system is needed to bring biomonitoring, environmental monitoring, disease burden data, exposure–response evidence, and policy evaluation into the same prevention framework. By making the health effects of pollution measurable, data science can help move chronic disease prevention upstream and support policies that reduce both environmental exposure and diabetes-related kidney disease burden.
